# Fluorescent cacao oil: A material suited for highlighting blood vessel in macro-scale dissection

**DOI:** 10.1016/j.mex.2022.101714

**Published:** 2022-04-27

**Authors:** Taketeru Tomita, Minoru Toda, Yuki Fukugawa, Takahide Sasai, Kiyomi Murakumo

**Affiliations:** a Okinawa Churashima Research Center, Okinawa Churashima Foundation; b Okinawa Churaumi Aquarium, Okinawa Churashima Foundation; c Tropical Dream Center, Okinawa Churashima Foundation; d Graduate School of Engineering and Science, University of the Ryukyus

**Keywords:** Imaging, UV light, Vascular visualization

## Abstract

This study describes a novel method to highlight vascular networks in animal tissue during macro-scale dissection using cacao oil and ultraviolet (UV) fluorescent dye. This is a three-step method: 1) injecting warmed cacao oil containing oil-based UV fluorescent dye (“fluorescent cacao oil” or FCO) into the blood vessels of a dead animal; 2) lowering the temperature to solidify the FCO in blood vessels; and 3) illuminating blood vessels with UV light when the specimen is dissected. This method uses the unique properties of cacao oil, which is solid at room temperature but becomes liquid at 40°C. Such a relatively low melting temperature meets two conflicting demands, i.e., maintaining low viscosity for better flow into the blood vessels and preventing damage of animal tissue by heat. This method is:

• Practical, as blood vessel is strongly highlighted using handy UV light during dissection; therefore, a specific medical equipment is not required
• Inexpensive, as FCO is made by mixing two commercially available produces (cacao oil and UV fluorescent dye)
• Stable, as FCO-injected tissue can be fixed and preserved semi-permanently in formalin. The fluorescent ability of FCO is not affected by this process

## Graphical abstract

**Figure:**
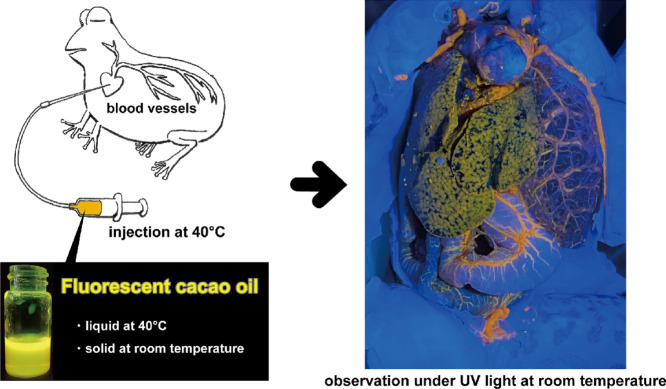

Specifications table

**Table:** 

Subject Area:	Agricultural and Biological Sciences
More specific subject area:	Anatomy
Method name:	Vascular highlighting with fluorescent cacao oil
Name and reference of original method:	Not applicable
Resource availability:	Not applicable

## Background

The vascular network in animal tissue is complex, and its structure is difficult to be grasped by macro-scale dissection. A simple solution is the injection of colored materials (e.g., ink, colored gelatin, and resin) into the blood vessel (reviewed in [1]). By this injection, the blood vessel route is highlighted, improving the visibility of blood vessels during dissection; however, in our experience, this method sometimes works insufficiently, especially when the background color of the tissues masks the resin color. Recently, this method has been used with computed tomography (CT). They add a radio-opaque contrast agent (e.g., barium sulfate) to a colored resin, inject it into a blood vessel, and take CT images before dissection [2,3]. Another well-known method is vascular corrosion casting (e.g., [4]); in this method, solidifying resin (latex in most cases) is injected into the blood vessel to make the blood vessel cast by dissolving surrounding tissue with corrosive chemicals, such as sodium hydroxide. While CT-scanning and vascular corrosion casting are powerful techniques for vascular visualization, there are some practical problems in the usage during dissection. The particular problem is that these methods do not allow real-time visualization during dissection. For example, vascular CT images are visible on the computer screen but not on the person's eyes performing the dissection. Similarly, in corrosion vascular casting, the cast of blood vessels can only be visualized after the surrounding tissues are dissolved.

Therefore, this study proposes a novel method to visualize blood vessels in the animal tissue using cacao oil mixed with UV fluorescent dye. Also, this method is advantageous compared with previous methods in its high visibility and ability of real-time visualization during dissection. Furthermore, this method has merit in non-requirement for expensive medical equipment or potentially hazardous chemicals.

## Method details

**Fig. 1:**
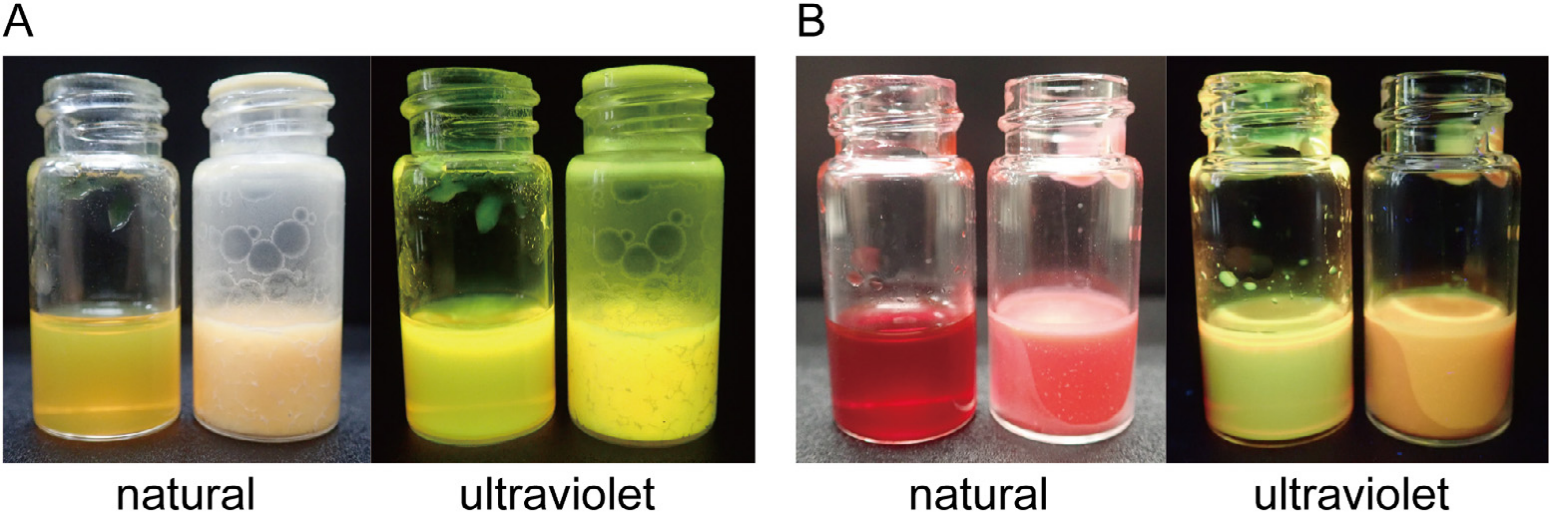
A. Fluorescent cacao oil (FCO) seen under natural and ultraviolet light. Left bottle includes liquid FCO; whereas right bottle includes solidified FCO. B. FCO colored with red oil-based ink seen under natural and ultraviolet light. Left bottle includes liquid FCO, whereas right bottle includes solidified FCO.

*Step 1*—Preparation of fluorescent cacao oil (FCO) (Fig. 1): The cacao oil is solid at room temperature; thus, it is warmed to be liquidized at a temperature of more than 40°C. After the cacao oil is completely melted, a small amount of oil-based UV fluorescent dye (0.2–0.3% of cacao oil in weight) is added to the cacao oil. The mixture of these is maintained at a temperature of more than 40°C to prevent it from solidifying. Optionally, FCO can be colored with oil-based color ink (Fig. 1B); however, adding a small amount of color ink does not prevent the solidification and fluorescence of FCO.

**Fig. 2:**
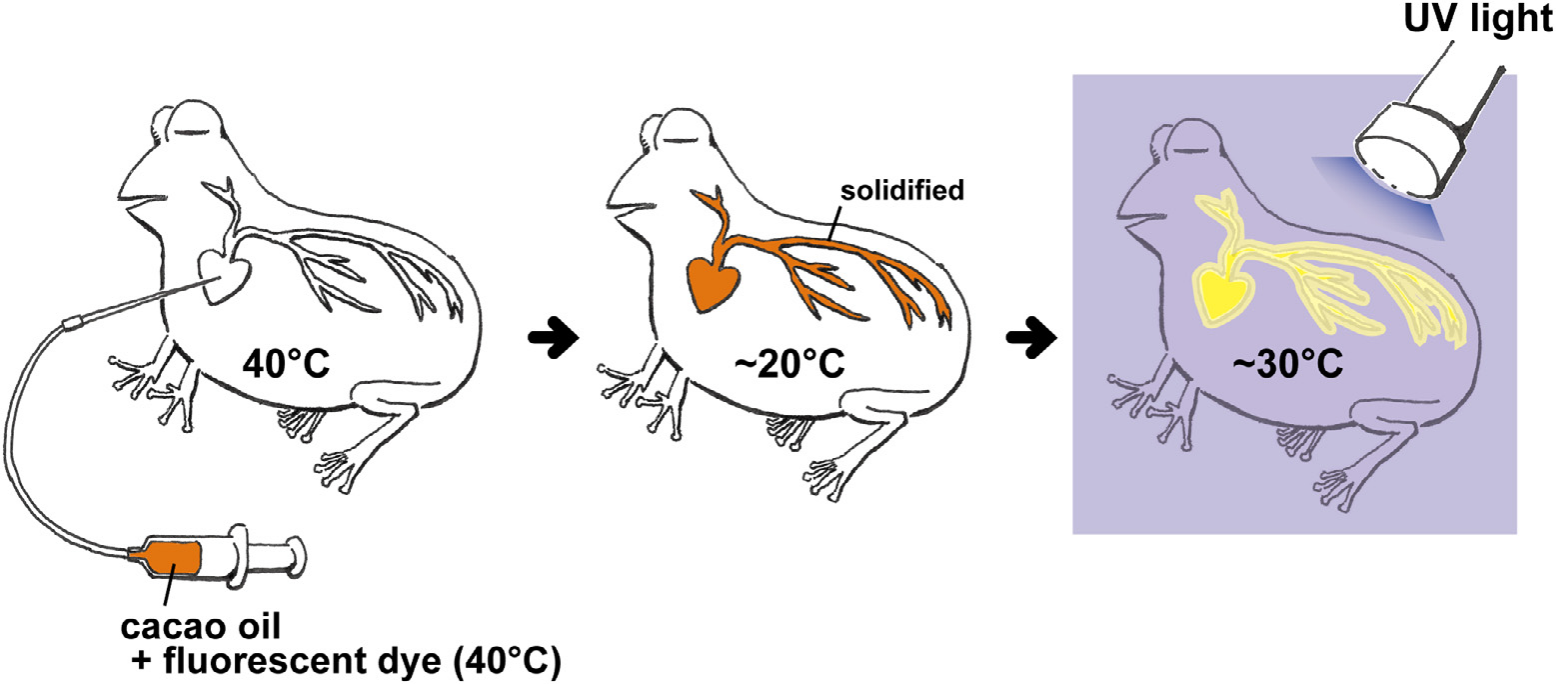
The process from injection of fluorescent cacao oil to vascular observation. Warmed (40°C) fluorescent cacao oil is injected into warmed (40°C) animal body (left). The animal body is then cooled (c.a. 20°C) to solidify the fluorescent cacao oil (middle). Finally, the animal body is fixed with formalin at ambient temperature (c.a. 30°C) and observed under UV light after dissection (right).

*Step 2*—FCO Injection (Fig. 2). The animal specimen is warmed to body temperature of ∼40 °C. This process prevents the FCO from clotting in blood vessels during the introduction. It should be noted that the warming temperature must not exceed 40 °C at which the heat damages the tissue protein. After FCO is fully introduced, the body temperature gradually decreases to ∼20 °C to solidify the FCO filling the blood vessels. Once FCO is solidified, it is maintained in the solid state at room temperature (about 30 °C). Rapid cooling (e.g., cooling in iced water) should be avoided because it causes the solidified cacao oil to be fragile. After cooling, the specimen is fixed with 5–10% formalin at room temperature.

*Step 3*—Observation. The specimen is cut open, and tissues are exposed in the normal procedure of dissection. By illuminating the tissue with UV light, the vascular network on the tissue surface is strongly highlighted.

## Additional notes to this method

FCO is soluble in alcohol. Three months exposure of FCO to 70% ethanol did not affect the condition of solidified FCO, though exposure beyond one year should be avoided.

## Example of the usage

### Materials

Two specimens of the bullfrog (*Lithobates catesbeianus*) were used in this study. These specimens were obtained from the invasive species-eradication program conducted by Okinawa Churashima Foundation at Ocean Expo Park (Okinawa, Japan). Furthermore, 0.4% phenoxyethanol was used to euthanize the specimens. Handing and euthanizing the animal was done in strict accordance with the guidelines for animal experiments of the Okinawa Churashima Foundation, with the same consideration for animal care and welfare as that for “higher” vertebrates (reptiles, birds, and mammals).

### Methods

The glass jar containing 50-g cacao oil was soaked in hot water at a temperature of 60°C. After the cacao oil was completely liquidized, 0.13-g UV fluorescent dye TP3400 (TRACER Products, Melville, NY) was mixed with the oil to make FCO. The cost of making 50-g FCO was approximately two US dollars. Next, the jar was placed in 40°C warm water to keep the FCO to be liquidized.

**Fig. 3:**
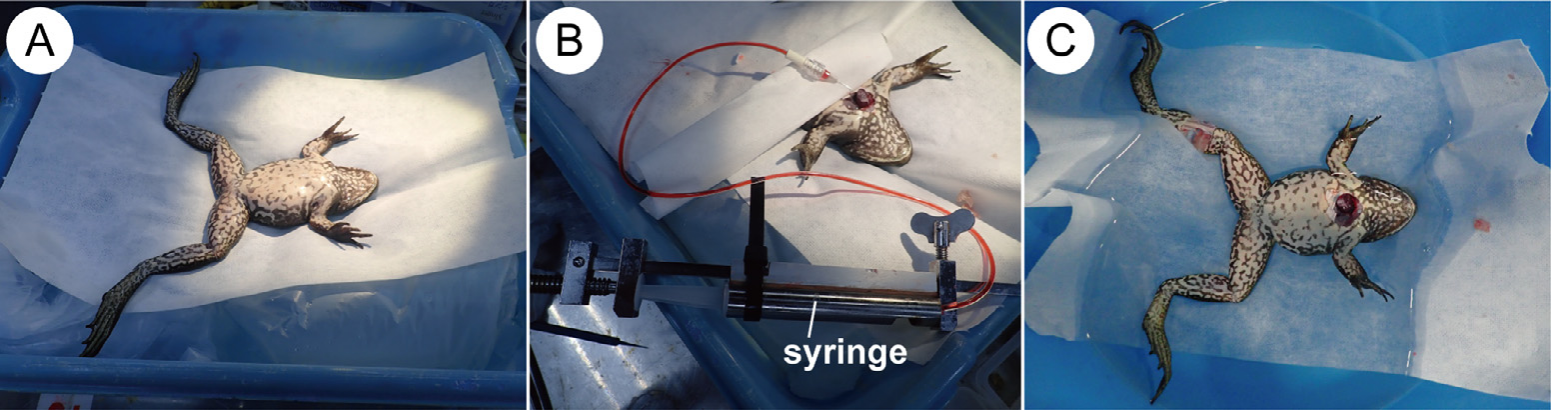
The process of fluorescent cacao oil injection for bullfrog. A) The specimen was warmed to 40°C on the water bed. B) Liquid vascular highlighter is injected into the specimen. C) After injection is completed, the specimen was cooled in 20°C water to solidify injected cacao oil.

Immediately after euthanizing the specimen, it was warmed up by placing it on the water bed filled with 40°C warm water (Fig. 3A), and its heart was exposed with surgical scissors. Next, liquid FCO is injected into the ventricle lumen with 5 ml syringe connected with an extension tube and 27-gage needle (Fig. 3B). The site was glued with Aron Alpha Super Jelly (Konishi Co., Osaka, Japan) to prevent FCO from leaking out of the needle puncture site. After 5 ml of FCO was introduced, the frog's body was soaked in water at a temperature of 20°C to make the FCO solidified (Fig. 3C). After 1 h of cooling, the animal body was fixed with 10% formalin at room temperature (25°C).

Two weeks later, we cut open the specimen to expose its internal organs by the regular dissection protocol. Then, in a dim light condition, we shined the internal organs of the specimen with UV penlight WUBEN E19UV (WUBEN Co., Hiroshima, Japan) to visualize blood vessels. Dissected specimens were photographed using an iPhone 11 camera (Apple Inc., Cupertino, CA) and a digital camera Tough TG-5 (Olympus Co., Tokyo, Japan).

### Results

**Fig. 4:**
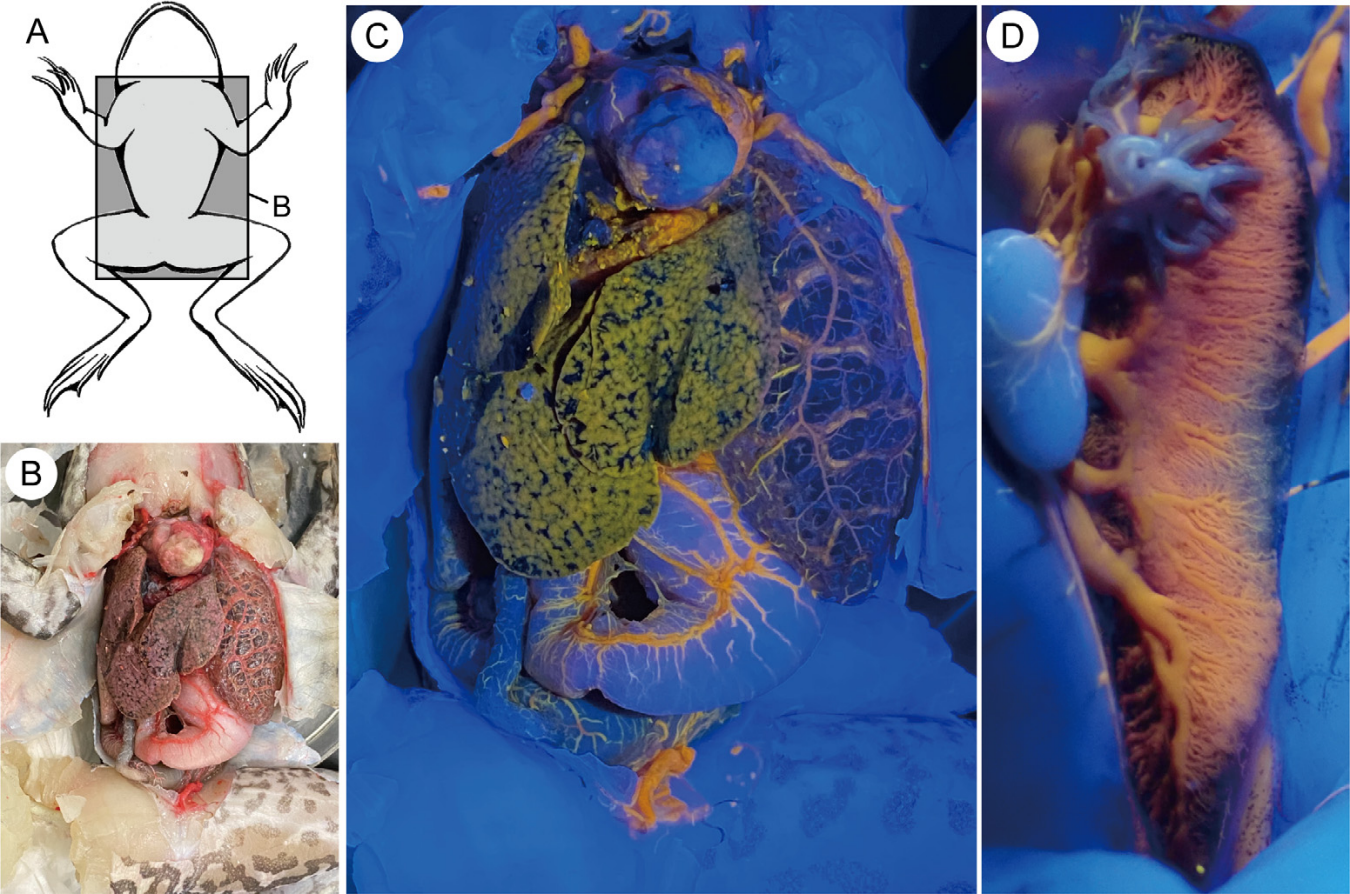
Examples of images obtained: A. Ventral view of bullfrog showing location of panel B. B. Dissected specimen photographed under normal light. C. Same location as B photographed under UV light. The blood vessels distributing on the internal organs are clearly visible. D. Kidney photographed under UV light. The branching blood vessels are seen.

The resulting photographs are shown in Fig. 4. Under UV light, blood vessels on the surface of the internal organs are strongly highlighted in yellow (Fig. 4C). According to the observation of the kidney under the optical microscope Leica M165C (Leica Microsystems, Germany) with UV light, we confirmed that fluorescent cacao oil was introduced into the very end of the branching blood vessels with a diameter of 70.9 μm (± 9.0 s.d., n=20).

## References

[1] Haenssgen K., Makanya A.N., Djonov V. (2014). Casting materials and their application in research and teaching. Microsc. Microanal..

[2] Holliday C.M., Ridgely R.C., Balanoff A.M., Witmer L.M. (2006). Cephalic vascular anatomy in flamingos (*Phoenicopterus ruber*) based on novel vascular injection and computed tomographic imaging analyses. Anat. Rec. A Discov. Mol. Cell. Evol. Biol..

[3] F.D. Verli, T.R. Rossi-Schneider, F.L. Schneider, L.S. Yurgel, M.A.L. de Souza, Vascular corrosion casting technique steps. Scanning 29 (2007) 128–132. 10.1002/sca.20051 17477397

[4] Costidis A., Rommel S.A. (2012). Vascularization of air sinuses and fat bodies in the head of the Bottlenose dolphin (*Tursiops truncatus*): morphological implications on physiology. Front. Physiol..

